# Perfectionism and Prospective Near-Term Suicidal Thoughts and Behaviors: The Mediation of Fear of Humiliation and Suicide Crisis Syndrome

**DOI:** 10.3390/ijerph17041424

**Published:** 2020-02-22

**Authors:** Tyler Pia, Igor Galynker, Allison Schuck, Courtney Sinclair, Gelan Ying, Raffaella Calati

**Affiliations:** 1 Department of Psychiatry, Mount Sinai Beth Israel, New York, NY 10003, USA; tsp2125@tc.columbia.edu (T.P.); Igor.Galynker@mountsinai.org (I.G.); schuckam13@gmail.com (A.S.); courtneymsinclair96@gmail.com (C.S.); gy2255@tc.columbia.edu (G.Y.); 2 Icahn School of Medicine at Mount Sinai, New York, NY 10029-6574, USA; 3 Department of Psychology, University of Milan-Bicocca, 20126 Milan, Italy; 4 Department of Adult Psychiatry, Nîmes University Hospital, 30029 Nîmes, France

**Keywords:** perfectionism, suicide/self-harm, fear of humiliation, psychiatric outpatients

## Abstract

*Background*: Perfectionism has been linked to suicide. According to the Narrative-Crisis Model of suicide, individuals with trait vulnerabilities are prone to develop a certain mindset, known as a Suicidal Narrative, which may precipitate the Suicide Crisis Syndrome (SCS), culminating in suicide. The purpose of this study was to investigate the association between perfectionism (trait vulnerability), fear of humiliation (component of the Suicidal Narrative), SCS, and prospective near-term suicidal thoughts and behaviors (STB). *Methods:* Adult psychiatric outpatient participants (*N* = 336) were assessed at baseline with the Suicidal Narrative Inventory for perfectionism and fear of humiliation. The questions used to assess perfectionism were adapted from the Multidimensional Perfectionism Scale. The severity of the SCS was calculated using the Suicide Crisis Inventory. STB were assessed at baseline and after one month using the Columbia Suicide Severity Rating Scale. Serial mediation analyses were conducted using PROCESS version 3.3 in SPSS. *Results*: While the direct effect of perfectionism on prospective STB was not significant (b = 0.01, *p* = 0.19), the indirect effect of perfectionism on STB, through serial mediation by fear of humiliation and the SCS, was significant (indirect effect *p* = 0.007, 95% CI [0.003, 0.013]). The indirect effect was not significant for models that did not include both mediators. *Limitations:* Variables were assessed at one time only. *Conclusion*: Perfectionism did not directly modulate STB. Perfectionism may be related to suicidal behavior through fear of humiliation, leading to the SCS. These results support the Narrative-Crisis Model of suicide and clarify the role of perfectionism in the etiology of suicide.

## 1. Introduction

In 2017, over 47,000 people died by suicide in the United States [1]. Suicide rates have been increasing remarkably, with an average increase of 33% across the United States from 1999 to 2016 [2]. During this time, suicide rates were increasing annually by 1% from 1999 through 2006, and 2% from 2006 through 2016.

There are many circumstances that could lead a person to contemplate suicide, and many models of suicide have been proposed. Many theories include predisposing trait vulnerabilities, which are risk factors that are thought to make a person more susceptible to suicide [3,4,5].

Specifically, perfectionism as a risk factor has been widely studied for its role in suicide [6,7,8]. The three most commonly discussed perfectionism dimensions are self-oriented, other-oriented, and socially prescribed perfectionism, and they each have been shown to have varying strengths of association with suicide risk [9,10,11,12]. Self-oriented perfectionism involves a person’s expectations for themselves, independent of other people’s expectations [5]. Self-oriented perfectionists set themselves to certain standards in order to meet their idea of “perfection”, although their expectations may be unrealistic and make them more prone to feelings of shame and guilt upon failure to meet these expectations [11,13]. Other-oriented perfectionism is observed when a person has unreasonably high expectations for other people, but not themselves. In this scenario, the person who has perfectionistic tendencies that is other-oriented expects others to be perfect, and is likely to find that people fail to meet their expectations. Research has not found a link between this dimension and suicide [7]. In the third dimension, socially prescribed perfectionism, people feel as though they are expected to be perfect by someone else [10,14]. People who exhibit this characteristic may feel compelled to perform at a certain standard because they feel obligated to by an external force, whether it be a cultural expectation, a societal expectation, or imposed upon them by significant people in their lives. This dimension has been the most consistently linked to suicidal ideation, past suicidal behaviors, and suicide potential [9,15,16,17,18].

Although trait vulnerabilities have long been studied for their role in suicidality, they do not on their own directly lead to near-term suicidal crisis [7]. Chronic trait vulnerabilities are often mediators or moderators of suicidal thoughts and behaviors (STB) when paired with more acute risk factors, such as negative life events or social problems [19,20]. Simply being a perfectionist does not directly lead to suicide, but the negative experiences caused by perfectionism have potential to be devastating. One particular acute negative experience that has been linked to both perfectionism and suicide is humiliation, both when a person perceives to have been humiliated and when a person has the fear of humiliation [21,22]. The combination of perfectionism and feelings of humiliation being implicated in suicidality is also present in O’Connor’s integrated Motivational-Volitional Model of suicidal behavior [19]. According to O’Connor, socially prescribed perfectionism is a vulnerability factor that increases the sensitivity to signals of defeat, while defeat/humiliation are the key drivers for the emergence of suicidal ideation. Perceived humiliation occurs when a person experiences negative affect after an event interpreted as humiliating. Fear of humiliation is the negative affect that is associated with the anticipation of a humiliating event or scenario, which occurs in the absence of an actual event. Rothstein (1984) describes fear of humiliation in the context of narcissistic personality disorder, in which any minor rejection or failure is perceived as a humiliation, and therefore, the person feels an inordinate need to be perfect [23]. The person fears that if he/she is humiliated, he/she will be imperfect and undeserving of love or wellbeing. If the person lives in fear that he/she will continue to experience humiliation, he/she is in danger of developing STB. It has been postulated that fear of humiliation can be so impactful that an individual may consider suicide just to avoid possible humiliation [24,25]. People who have perfectionistic traits are more self-critical concerning their mistakes, and are, therefore, more likely to interpret unsuccessful situations as humiliating [5].

Perfectionism and fear of humiliation are part of a comprehensive empirically based Narrative-Crisis Model of suicide recently developed by Galynker and coworkers [26]. The model was partially validated in previous reports [27,28,29,30], and was fully supported by the Structural Equation Modeling analysis [31]. The Narrative-Crisis Model of suicide is similar to other models of suicide in that it incorporates the elements of the Interpersonal Theory of Suicide, the already mentioned Motivational-Volitional Model and the Stress-Diathesis Model, which have previously received experimental support. The specific elements are O’Connor’s Entrapment [19] and Joiner’s Perceived Burdensomeness and Thwarted Belongingness [32]. The Narrative-Crisis Model differs from other models in that it distinguishes between long-term and short-term risk factors and does not include suicidal ideation as risk factor. Onset of conscious suicidal ideation could be minutes before suicide attempt or never [33], and many suicidal patients do not disclose their suicidal ideation to clinicians [34]. Further, the Narrative-Crisis Model differs from other models in that it includes a suicide-specific diagnosis of Suicide Crisis Syndrome (SCS) [35,36], and that it uses the narrative identity-based concept of Suicidal Narrative [26].

**Figure 1:**
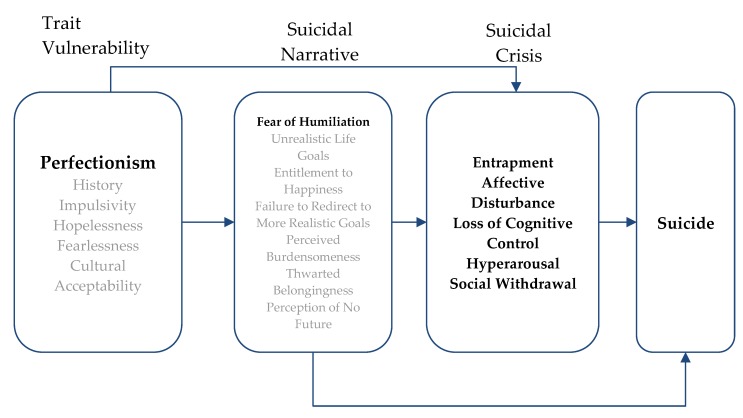
The Narrative-Crisis Model of suicide.

The Narrative-Crisis Model is comprised of three components that could lead to suicide: trait vulnerabilities, the Suicidal Narrative, and the SCS. Past studies on components of the Narrative-Crisis Model have shown that components of the SCS and the Suicidal Narrative in conjunction with vulnerability traits are associated with STB [27,29,30]. See Figure 1 for an explanation of how the Narrative-Crisis Model components interact to predict suicide. Trait vulnerabilities, as mentioned earlier, have been reported as long-term risk factors for suicidal behavior; according to the Narrative-Crisis Model, at least one trait vulnerability should be present in order to be susceptible to the Suicidal Narrative or the SCS. Trait vulnerabilities include a person’s history of adverse experiences, impulsivity, hopelessness, fearlessness, cultural acceptability, and, finally, perfectionism.

A Suicidal Narrative is a disruption in the narrative identity [37] that a person develops over time, according to which he/she believes that suicide is the only option [26] and may give rise to the SCS [27]. This narrative identity disruption puts the individual at risk for suicide. The Suicidal Narrative is comprised of seven components that add up to a storyline, which represents the way a person perceives their past experiences and expects their future to unfold. In this storyline, people perceive that their lives are unbearable and that their future circumstances will never improve. The narrative identity of the Suicidal Narrative entails setting up unrealistic life goals, feeling entitled to happiness when and only when these goals are achieved, and being unable, for reasons of entitlement, to disengage from these unattainable goals and unable to redirect to more realistic ones. The Suicidal Narrative continues with what is perceived as humiliating personal or social defeat in failing to achieve the unachievable goals, which result in the perception of burdensomeness (Perceived Burdensomeness) to more successful others, the perception of not belonging to others (Thwarted Belongingness), and, finally, the perception of no future. Perceived Burdensomeness and Thwarted Belongingness are present in Joiner’s Interpersonal Theory of suicide; according to his model, the presence of these two constructs would lead to the emergence of suicidal ideation [32]. The Suicidal Narrative is hypothesized to be primarily a cognitive construct that is mutually reinforced with the primarily affective state of SCS.

The SCS is a condition that occurs before a suicide attempt, in which a person experiences an acute, negative cognitive and emotional state. The SCS consists of five symptoms, which are a feeling of entrapment, affective disturbance, loss of cognitive control, hyperarousal, and social withdrawal [35]. The presence of these symptoms is predictive of an imminent suicide attempt [38].

In this study, we aimed to test whether trait perfectionism could predict prospective near-term STB when mediated by fear of humiliation and the SCS severity in a serial mediation model. This study is the first to examine multi-stage mediation of near term STB. We tested each component of perfectionism (self-oriented, other-oriented, and socially prescribed) as well as perfectionism as a whole. We hypothesized that the trait of perfectionism mediated by fear of humiliation and SCS severity, at baseline, could predict STB at one-month follow up.

## 2. Methods

### 2.1. Subjects

Participants were 336 psychiatric outpatients who were referred to the study by their clinician at a large, urban hospital. Participants were referred upon their initial visit with a clinician for any psychological or psychosocial treatment. The referring clinician determined whether a patient met exclusion criteria during the initial visit. Participants were only eligible for participation if they were able to understand and sign a consent form after being informed about the study, its aims, its risks, and its benefits. Inclusion criteria for the study were met if participants: were at least 18 years of age, were domiciled, were English-speaking, presented for initial appointment for any pharmacological or psychosocial treatment, were able to understand the informed consent, and were able to provide at least two verifiable contacts to improve tracking for the subsequent assessments. Exclusion criteria for the study included active psychosis, agitation, mania, and/or cognitive deficits that made participants unable to give informed consent or unable to complete the tasks of the study. The present sample is part of a larger one recruited for the Modular Assessment of Risk for Imminent Suicide (MARIS) project. The study was approved by Mount Sinai Beth Israel’s Institutional Review Board (IRB) for Human Subjects Research.

### 2.2. Measures

#### 2.2.1. Baseline Measures

*Multidimensional Perfectionism Scale (MPS).* The MPS is a 45-item scale that measures the three dimensions of perfectionism: self-oriented, other-oriented, and socially prescribed [39]. The MPS has been shown to have adequate levels of reliability and validity in clinical samples [40]. Questions from this scale are present in the Suicidal Narrative Inventory (SNI), which was the scale administered in the present study. The questions have been adapted to be consistent with the other questions in the SNI.

*Suicidal Narrative Inventory (SNI).* The SNI measures the 7 components of the Suicidal Narrative. Fear of humiliation was measured using the fear of humiliation subscale of the Humiliation Inventory in the SNI. Trait perfectionism was measured using the adapted MPS questions in the SNI. The SNI rating scale goes from 1 (not at all true) to 5 (extremely true). The humiliation subscale has been shown to have high inter-item consistency [27].

*Suicide Crisis Inventory (SCI).* The SCI assesses SCS symptoms. It is a self-report questionnaire consisting of 49 items. Participants were asked to rate their experiences with symptoms over the last several days on a scale from 0 (not at all) to 4 (extremely). The SCI is a measure used to evaluate the severity of an acute state that may precede suicidal behaviors, and has high internal consistency and predictive validity [41].

*Affective Intensity Rating Scale (AIRS).* The AIRS assesses depressive turmoil. Participants were asked to rate their experiences with an affect over the last three days on a scale from 0 (not at all) to 4 (extreme). The AIRS has been shown to have internal consistency, as well as convergent and divergent validity when regressed against the Brief Symptom Inventory (BSI) [42].

*Brief Symptom Inventory (BSI).* Five items from the BSI were used to measure agitation, frantic anxiety, irritability, and hypervigilance. Participants were asked to rate their feelings over the past week on a scale from 1 (not at all) to 5 (extremely). The BSI has been shown to have high internal consistency and test-retest reliability [43].

*Beck Depression Inventory (BDI).* The BDI was used to measure depressive symptoms, specifically acute anhedonia [44]. Participants were asked to rate their depressive symptom severity over the past week using a Likert scale ranging from 0 (no symptom) to 3 (severe symptom). Acute anhedonia was measured using 2 items. The BDI has been shown to have high internal consistency, strong construct validity, and high concurrent validity when compared to other measures of depression [45].

*Visual Analog Scale (VAS) on social connectedness.* A 2 item VAS was used to measure feelings of social connectedness. Each item consisted of 5 pairs of circles at increasing distances, with scores ranging from 0 to 4. Circles that were completely separated corresponded to a score of 0, and circles that encompassed each other completely corresponded to a score of 4. In item 1, participants were asked to choose the pair of circles that best describes their relationship with their family, friends, and support network. In item 2, participants were asked to choose the pair of circles that best described their relationship with society. VAS measures have been found to have good validity and reliability across multiple settings [46,47].

#### 2.2.2. One Month Follow-Up Measures

*Columbia Suicide Severity Rating Scale (C-SSRS).* The C-SSRS is the gold standard to assess suicidal ideation and suicidal behavior. It is a semi-structured interview that entails asking participants whether they have experienced suicidal thoughts or engaged in suicidal behaviors or actions. Ideation has a rating scale of 1 to 5. The thoughts that are present are further rated by frequency, duration, controllability, whether the person had deterrents that stopped them from acting on thoughts, and reasons for ideation. For suicidal behaviors, participants were asked whether they have engaged in preparatory acts for suicide, non-suicidal self-injurious behavior, actual attempts, interrupted attempts, or aborted attempts. Lethality is also screened. We calculated a continuous variable for STB similar to the methodology of previous publications [36]. STB follows a 10-point scale, with scores 1–5 representing suicidal ideation and scores 5–10 representing suicidal behaviors. A score of 5 or higher indicates that a person is at risk of suicide. The C-SSRS has been shown to have good predictive validity and internal consistency [48].

### 2.3. Statistical Analyses

**Figure 2:**
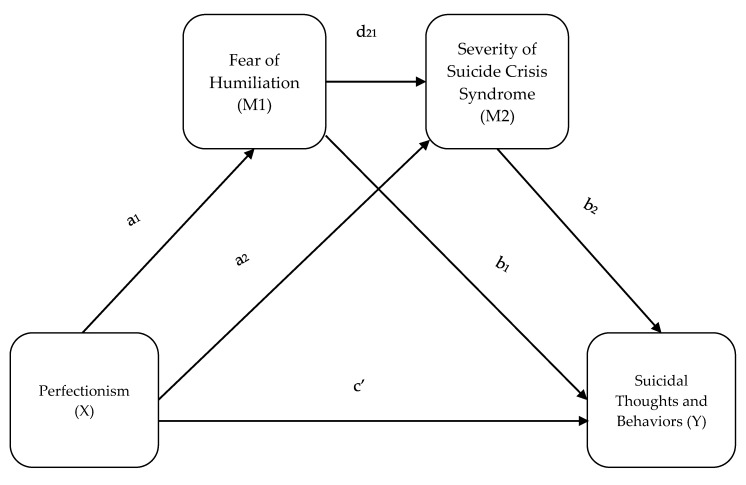
Serial multiple mediation schema.

Statistical analyses were conducted using the IBM Statistical Package for the Social Sciences (SPSS), version 25 (IBM Corp., Armonk, NY, USA). We assessed the normality distribution of each continuous variable using the Shapiro–Wilk test. Since all the variables were not normally distributed, we performed non-parametric tests, in particular the Spearman rho correlation. Mediation analyses were performed using PROCESS version 3.3 in SPSS. Tests were run to determine the direct effect of the predictor variable (perfectionism) on the outcome variable (STB), as well as the indirect effects of perfectionism with and without the mediating variables (fear of humiliation and SCS severity). A serial mediation was conducted using the dimensions of perfectionism that were significantly correlated with STB as the x-variables in order to incrementally test the relationships between all of the variables. STB at 1 month follow up was the y-variable, and fear of humiliation and SCS severity were mediators. The model of this study follows the structure of a serial multiple mediation with two mediators, as displayed in Figure 2.

## 3. Results

### 3.1. Descriptive Statistics

The sample was composed of 336 participants. Demographic information can be seen in Table 1. The mean age of the patients was 39.01 ± 14.25 years old (age range: 18–84). The gender rate was 66.4% female, 30.7% male, and 3% other. The mean age of female participants was 38.78 ± 14.45 (age range: 18–84), the mean age of male participants was 40.43 ± 12.81 (age range: 18–71), and the mean age of participants who identified as “other” was 29.60 ± 11.07 (age range: 21–59). The mean years of education were 14.68 ± 3.10. The most prevalent primary diagnosis was depressive disorder (*n* = 150, 44.6%). The sample was racially diverse, being 38.7% white, 28.3% other, 24.7% black, and 8.3% Asian. “Other” represented participants who identified as multiracial or did not identify with the other categories. At 1 month follow up, 13 participants (3.9%) were classified as at high suicide risk: 2 participants (0.6%) endorsed SI with specific plan and intent, 5 participants (1.5%) reported making preparatory acts for a suicide attempt (e.g., writing a suicide note or giving away possessions), 4 participants (1.2%) reported that they were about to make a suicide attempt but ultimately aborted the attempt, and 2 participants (0.6%) reported making a suicide attempt that had low lethality. There were no reported deaths by suicide at 1 month follow up.

### 3.2. Binary Relationships

Spearman rho correlations were calculated between 7 variables: self-oriented perfectionism, other-oriented perfectionism, socially prescribed perfectionism, perfectionism as one combined variable, fear of humiliation, severity of the SCS, and STB at 1 month follow up.

The only dimension of perfectionism that was significantly correlated with STB at 1 month follow up was socially prescribed perfectionism. The combined variable of perfectionism was not significantly correlated with STB at 1 month follow up. Socially prescribed perfectionism (r = 0.16, *p* = 0.003), fear of humiliation (r = 0.14, *p* = 0.01), and the SCS severity (r = 0.30, *p* < 0.001) were significantly correlated with STB at 1 month follow up.

There are a number of other variables that were significantly correlated with each other (Table 2). Predictably, the different dimensions of perfectionism were significantly correlated with each other and with the combined perfectionism dimensions (all *p* < 0.001). Self-oriented, socially prescribed, and combined perfectionism were positively correlated with fear of humiliation (all *p* < 0.001), while other-oriented perfectionism was not correlated with fear of humiliation. Self-oriented, socially prescribed, and combined perfectionism were positively correlated with the SCS severity (*p* < 0.05, *p* < 0.001, and *p* < 0.001, respectively). Socially prescribed perfectionism, fear of humiliation, and the SCS severity were all positively correlated with the presence of STB at 1 month follow up (*p* = 0.003, *p* = 0.01, and *p* < 0.001, respectively). A mediation model was then fit to show the relationships between socially prescribed perfectionism, fear of humiliation, SCS severity, and STB at 1 month follow up.

### 3.3. Serial Mediation

Variables that were significantly correlated with the presence of STB at 1 month follow up were included in the mediation model. The variables included were socially prescribed perfectionism, fear of humiliation, and SCS severity. The model of this study follows the structure of a serial multiple mediation with two mediators, as displayed in Figure 2.

**Figure 3:**
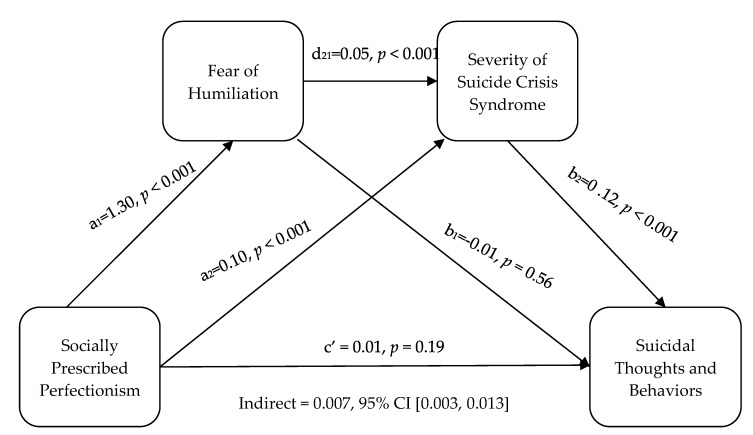
The relationship among the variables in the serial multiple mediation analysis.

The direct effect of socially prescribed perfectionism on STB was not significant (b = 0.01, t = 1.30, *p* = 0.19), meaning that, when controlling for fear of humiliation and SCS severity, socially prescribed perfectionism did not predict STB, although there was a significant, positive correlation between socially prescribed perfectionism and STB. The total effect of socially prescribed perfectionism on STB when mediation variables were not included in the model was significant (b = 0.03, t = 3.27, *p* = 0.001). The serial mediation model in which socially prescribed perfectionism leads to fear of humiliation, which leads to SCS, and which predicts STB at 1 month follow up, was significant (indirect effect = 0.007, 95% CI [0.003, 0.013]). The relationships between each of the variables can be seen in Figure 3.

According to the model, there were significant relationships between socially prescribed perfectionism and fear of humiliation (b = 1.30, t(334) = 12.56, *p* < 0.001), socially prescribed perfectionism and SCS severity (b = 0.10, t(333) = 4.58, *p* < 0.001), and SCS severity and STB at 1 month follow up (b = 0.12, t(332) = 4.25, *p* < 0.001). The relationship between socially prescribed perfectionism and STB (b = 0.01, t(332) = 1.30, *p* = 0.19) and fear of humiliation and STB (b = −0.01, t(332) = −0.59, *p* = 0.56) were not significant. Hence, socially prescribed perfectionism and future STB were related, but the relationship was not significant unless mediated by fear of humiliation and SCS severity.

## 4. Discussion

The aim of this study was to determine whether near-term suicidal thoughts and behaviors (STB) could be prospectively predicted by perfectionism, fear of humiliation, and the Suicide Crisis Syndrome (SCS) according to the Narrative-Crisis Model of suicide. Two dimensions of perfectionism, self-oriented perfectionism and socially prescribed perfectionism, were found to be significantly, positively correlated with fear of humiliation. This finding is consistent with similar findings reporting that self-oriented and socially prescribed perfectionism are correlated with fear of shame and embarrassment, and greater feelings of shame and guilt [13,49]. The serial mediation showed that both fear of humiliation and the SCS significantly mediated the relationship between socially prescribed perfectionism and the STB at 1 month follow up. Socially prescribed perfectionism was found to be associated with STB at 1 month follow up only when fear of humiliation and SCS were included in the serial mediation model. Literature linking perfectionism and suicidal ideation is extensive [6,7], and many studies found that the association between perfectionism and STB is better explained when there is some moderating or mediating factor involved, such as hopelessness or environmental stressors [50]. Having perfectionistic tendencies can make a person more susceptible to maladaptive behaviors, for example “all or nothing” thinking, which can cause people to feel as though their failures are catastrophic. In such instances, people may even consider suicide because they feel as though they have failed at their lives completely [51]. In both the present study and the Conroy et al. study, socially prescribed perfection was found to be the most highly correlated with fear of humiliation when compared to self-oriented and other-oriented perfectionism, which is further consistent with the theory that the experience surrounding humiliation, including fear of humiliation and fear of failure, is influenced by the presence and perceived judgements of others [49,52]. The fact that the analysis was significant only when both socially prescribed perfectionism and fear of humiliation were included supports the Narrative Crisis Model, which is an example of a trait vulnerability leading to a narrative state, and then leading to a suicide crisis, which then leads to prospective near-term STB. The direct effect of socially prescribed perfectionism on STB was not significant, further supporting the theory that it is the combination of socially prescribed perfectionism, fear of humiliation, and SCS, rather than any of these factors alone, that plays a role in a person’s STB. In the same vein, other studies found perfectionism to be a risk factor for STB, but it did not have a direct link with STB [53,54]. In this specific study, the progression of events leading to STB would be as follows: a person who experiences socially prescribed perfectionism is prone to find him or herself in a state of fearing humiliation, which then can lead to the SCS, in which a person feels as though they are trapped or somehow losing control of themselves, which then leads to STB.

It is also worth noting that the only dimension of perfectionism that had a significant correlation with STB was socially prescribed perfectionism, which is related to high expectations imposed by others. When combining all three dimensions into one variable, the correlation between perfectionism and STB was not significant. There has been evidence that socially prescribed perfectionism is the dimension that is most related to feelings of inadequacy, entrapment, and loneliness [18,55,56]. Tangney suggested that this may occur because self-oriented perfectionists feel as though they choose which domains of their lives they must be perfect in and, therefore, are able to better manage their own expectations [5]. With socially prescribed perfectionists, individuals perceive that they have little or no control over what is expected from them, and when they cannot meet these expectations, which they did not feel they could accomplish in the first place, they are more likely to feel hopeless, isolated, and trapped. Recognizing that the combination of socially prescribed perfectionism and fear of humiliation can lead to the SCS and then imminent STB may help clinicians to detect people who are at risk without reliance on the self-report of suicidal ideation, which is often unreliable and could be misleading [52].

This study has a number of strengths. First, the study is rare in its prospective design and its focus on near-term suicide outcomes. Second, the study has a relatively large sample size. Third, rather than treating perfectionism as a single variable, the SNI assessed its three distinct dimensions allowing a more nuanced analysis of the relationship between different types of perfectionism and future near-term suicidal behaviors.

Our study has limitations as well. Perfectionism and fear of humiliation were measured using the same scale (SNI), although according to the Narrative-Crisis Model, they represent constructs with different time frames [25]. Perfectionism is thought to be a trait, which is unchanging and stable across a person’s lifetime. Fear of humiliation is thought to be a narrative state, which is not fixed. Ideally, these two variables should be assessed using measures that reflect the differences between a fixed trait and a narrative state. Participants were only assessed at one point for perfectionism, fear of humiliation, and SCS, so it is also not possible to establish directionality of the variables. Another limitation is that we have only assessed fear of humiliation, which is a rather narrow concept. Future exploration of the relationship between humiliation and suicide should include early and past experiences of humiliation and the actual experience of humiliation in order to give a more comprehensive description of the narrative state of humiliation that a person is experiencing.

Within the study limitations, present findings further add to the research on the predictive power of the Narrative-Crisis Model of suicide and the role of perfectionism and fear of humiliation in STB. The theoretical link between perfectionism and fear of humiliation has been explored, and this study adds to the small pool of empirical evidence that exists for this connection. In addition, this is the first study to connect perfectionism, fear of humiliation, SCS, and STB in a mediation model. This study was conducted using a sample of psychiatric outpatients, but because trait perfectionism and fear of humiliation are not experiences unique to a clinical population, we hypothesize that the findings may be generalized to non-clinical populations as well. Future research can focus on the directionality of the components of the Narrative-Crisis Model, and whether the inclusion of more vulnerability traits and/or multiple narratives is better predictive of STB. Moreover, further research on this topic may benefit from conducting a longitudinal study with multiple follow-ups in order to get a better sense of whether this model is predictive of future STB. The Narrative-Crisis Model provides a basic framework for assessing trait vulnerabilities and narratives that may lead a person to STB. The model allows us to better understand the cognitive distortions of the mindset of a person at imminent risk of suicide so better methods could be designed for its replacement for more positive life narratives.

## 5. Conclusions

Perfectionism did not directly modulate suicidal thoughts and behaviors in a sample of adult psychiatric outpatient participants. However, it may be related to suicidal behavior through fear of humiliation, leading to the SCS. These results support the Narrative-Crisis Model of suicide and may help to clarify the role of perfectionism in the etiology of suicide.

## Figures and Tables

**Table 1:** Sociodemographic and clinical features of the sample (*N* = 336).

Variables *		Mean [SD] or *N* (%)
Age		39.01 [14.25]
Gender	Male	103 (30.7)
	Female	223 (66.4)
	Other	10 (3.0)
Race	Asian	28 (8.3)
	Black	83 (24.7)
	White	130 (38.7)
	Other	91 (28.3)
Years of Education		14.68 [3.10]
Primary Diagnosis	Depressive disorder	150 (44.6)
	Anxiety disorder	38 (11.3)
	Bipolar and related disorders	41 (12.1)
	Schizophrenia spectrum	23 (6.8)
	Obsessive-compulsive disorders	1 (0.3)
	Trauma and stress-related disorders	43 (12.8)
	Other	16 (4.8)

* Missing data: Years of education: 5; Ethnicity: 1; Race: 4; Marital Status: 1; Annual income: 6; Primary diagnosis: 1.

**Table 2:** Spearman rho correlations between Perfectionism, Fear of Humiliation, Severity of the Suicide Crisis Syndrome, and Suicidal Thoughts and Behaviors at 1 month follow up.

Variables	Self-Oriented Perfectionism	Other-Oriented Perfectionism	Socially Prescribed Perfectionism	Fear of Humiliation	Suicide Crisis Syndrome Severity	Suicidal Thoughts and Behaviors at 1 Month Follow-Up
	Spearman rho, *p* value					
Self-Oriented Perfectionism		0.32, <0.001	0.46, <0.001	0.30, <0.001	0.11, 0.05	NS
Other-Oriented Perfectionism	0.32, <0.001		0.20, <0.001	NS	NS	NS
Socially Prescribed Perfectionism	0.46, <0.001	0.20, <0.001		0.58, <0.001	0.45, <0.001	0.16, 0.003
Fear of Humiliation	0.30, <0.001	NS	0.57, <0.001		0.46, <0.001	0.14, 0.01
Suicide Crisis Syndrome Severity	0.11, 0.05	NS	0.45, <0.001	0.46, <0.001		0.14, 0.01
Suicidal Thoughts and Behaviors at 1 Month Follow-Up	NS	NS	0.16, 0.003	0.14, 0.01	0.30, <0.001	

(NS: Not Significant).
